# Mechanism and regulation of DNA damage recognition in nucleotide excision repair

**DOI:** 10.1186/s41021-019-0119-6

**Published:** 2019-01-25

**Authors:** Masayuki Kusakabe, Yuki Onishi, Haruto Tada, Fumika Kurihara, Kanako Kusao, Mari Furukawa, Shigenori Iwai, Masayuki Yokoi, Wataru Sakai, Kaoru Sugasawa

**Affiliations:** 1 Biosignal Research Center, Kobe University, 1-1 Rokkodai-cho, Nada-ku, Kobe, Hyogo 657-8501 Japan; 2 Graduate School of Science, Kobe University, 1-1 Rokkodai-cho, Nada-ku, Kobe, Hyogo 657-8501 Japan; 3 Faculty of Science, Kobe University, 1-1 Rokkodai-cho, Nada-ku, Kobe, Hyogo 657-8501 Japan; 4 Graduate School of Engineering Science, Osaka University, 1-3 Machikaneyama-cho, Toyonaka, Osaka, 560-8531 Japan

**Keywords:** Nucleotide excision repair, DNA damage recognition, XPC, TFIIH, XPA, UV-DDB, Chromatin

## Abstract

Nucleotide excision repair (NER) is a versatile DNA repair pathway, which can remove an extremely broad range of base lesions from the genome. In mammalian global genomic NER, the XPC protein complex initiates the repair reaction by recognizing sites of DNA damage, and this depends on detection of disrupted/destabilized base pairs within the DNA duplex. A model has been proposed that XPC first interacts with unpaired bases and then the XPD ATPase/helicase in concert with XPA verifies the presence of a relevant lesion by scanning a DNA strand in 5′-3′ direction. Such multi-step strategy for damage recognition would contribute to achieve both versatility and accuracy of the NER system at substantially high levels. In addition, recognition of ultraviolet light (UV)-induced DNA photolesions is facilitated by the UV-damaged DNA-binding protein complex (UV-DDB), which not only promotes recruitment of XPC to the damage sites, but also may contribute to remodeling of chromatin structures such that the DNA lesions gain access to XPC and the following repair proteins. Even in the absence of UV-DDB, however, certain types of histone modifications and/or chromatin remodeling could occur, which eventually enable XPC to find sites with DNA lesions. Exploration of novel factors involved in regulation of the DNA damage recognition process is now ongoing.

## Background

Genomic DNA constantly suffers from damage caused by a wide variety of agents from endogenous as well as environmental sources. Such DNA damage can interfere with normal processes of DNA replication, transcription, and chromosome segregation, thereby inducing genomic instability, cellular senescence, and/or apoptosis. As the primary defense system against these deleterious effects, organisms have evolved multiple DNA repair pathways.

Nucleotide excision repair (NER) is a major DNA repair pathway, which can eliminate various helix-distorting DNA lesions that are generated mainly by environmental mutagens, such as ultraviolet light (UV) irradiation and bulky chemical compounds [1]. In humans, hereditary defects in NER have been implicated in several autosomal recessive disorders, such as xeroderma pigmentosum (XP), Cockayne syndrome, trichothiodystrophy, and UV-sensitive syndrome. Eukaryotic NER consists of two sub-pathways: global genomic NER (GG-NER) and transcription-coupled NER. The former is particularly important for suppression of UV-induced mutagenesis and carcinogenesis, as revealed by a marked predisposition to skin cancer associated with patients of XP [2].

In general, DNA damage recognition is the first key step, which affects overall efficiency of DNA repair. Concerning mammalian GG-NER, two XP-related gene products, XPC and DDB2 (XPE), play central roles in the damage recognition process [3]. Following the initial lesion detection, verification of the presence of relevant lesions is also crucial for ensuring accuracy of the entire repair system, in which the transcription factor IIH (TFIIH) and XPA are involved. In addition, it remains to be understood how chromatin structures around sites of DNA damage affect damage recognition and are altered to allow initiation of the repair process.

## Lesion recognition and verification in GG-NER

In mammalian GG-NER, the XPC-RAD23-CETN2 heterotrimer plays a pivotal role in lesion recognition [4–6]. This protein complex can detect and bind DNA sites, where the regular double-helical structure is perturbed and, as a result, one or more base pairs are disrupted and/or destabilized [7–9]. Because any specific lesion structure is not the issue, such unique DNA binding properties of XPC underlie the infinite spectrum of substrate specificity exhibited by GG-NER. Recent studies suggest that XPC interrogates intactness of DNA structures mainly by a 1D-diffusion mechanism [10], and the presence of a helical distortion allows longer retention of XPC at the suspicious site and lowers an energy barrier that has to be overcome to form a stable DNA-protein complex [11].

As a nature of such an indirect damage sensor, XPC can bind to DNA sites containing only mismatched bases, but devoid of any lesion. In order to avoid erroneous incisions at such damage-free sites, it is essential for GG-NER to verify that a relevant lesion indeed exists. Accumulating evidence indicates that this is accomplished by the DNA-dependent ATPase/helicase activities of TFIIH in concert with XPA [12–14]. TFIIH contains two ATPase/helicase subunits, XPB and XPD [15, 16], and the XPB ATPase is essential for both transcription and NER [17]. Although XPB in vitro exhibits a relatively weak helicase activity with a 3′-5′ polarity, this helicase is dispensable for NER unlike its ATPase activity [18]. On the other hand, XPD has ATPase and 5′-3′ helicase activities, which are required for NER, but not for transcription [19, 20].

**Fig. 1:**
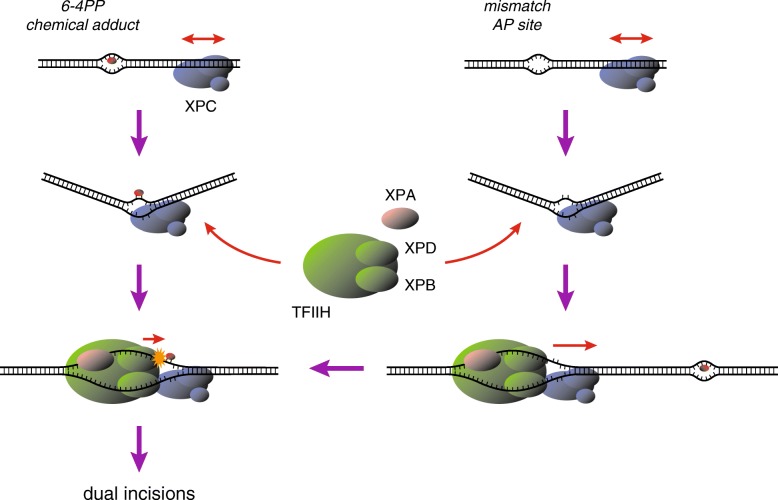
A model for DNA damage recognition and verification in mammalian GG-NER. The XPC protein complex recognizes and binds to DNA sites with disrupted/destabilized base pair(s) regardless of the presence (*left*) or absence (*right*) of relevant lesions. After loading of the TFIIH complex, the XPD helicase subunit in TFIIH scans a DNA strand in 5′-3′ direction in conjunction with XPA, and the presence of a lesion is finally verified by blockage of this translocation. Even though a lesion is not present exactly at the XPC-bound site, this scanning mechanism may provide NER with a chance to find some nearby lesions

We have previously shown with the cell-free NER system that the presence of mismatched bases supports efficient DNA binding by XPC and stimulates subsequent dual incisions. This effect is obvious especially with UV-induced cyclobutane pyrimidine dimers (CPDs), which induce only a small distortion of the DNA duplex and thus tend to escape detection by XPC [8]. Biochemical analyses revealed that XPC has to interact with unpaired bases on the undamaged DNA strand in order to induce productive dual incisions [14]. Importantly, a lesion to be incised can be apart from the XPC-binding site up to hundreds of bases, while dual incisions occur only when XPC binds to the 5′-side (but not 3′-side) of the lesion. Based on these findings, we have proposed that, after XPC detects and interacts with widowed bases, TFIIH is recruited such that the XPD helicase is loaded on the opposite DNA strand and translocates along it in a 5′-3′ direction [14] (Fig. 1). On the other hand, there have been reports from several groups indicating that the 5′-3′ helicase activities of yeast and archaeal XPD homologs as well as the intact human TFIIH complex are inhibited by the presence of bulky DNA lesions [12, 13, 21]. Taken together, the presence and location of relevant lesions are verified presumably by blockage of the XPD helicase translocation, thereby licensing the intermediate repair complex to proceed toward dual incisions (Fig. 1). It is also notable that XPA enhances not only the TFIIH helicase activity, but also its inhibition by DNA lesions [12]. XPA exhibits specific binding affinity for damaged DNA, especially some structures containing sharply kinked DNA strands [22, 23]. Therefore, interaction with XPA may modulate enzymatic and/or structural properties of the TFIIH helicase and thus affect its movement across the lesion sites.

**Fig. 2:**
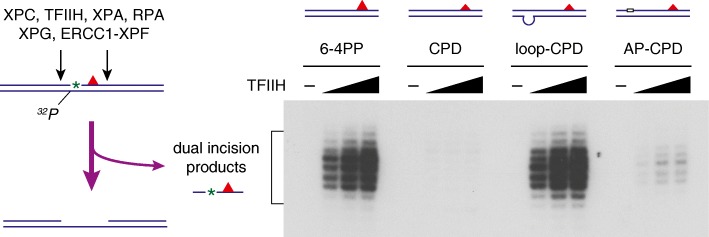
Stimulation of in vitro dual incision reactions with CPD substrates by the presence of mismatched bases or an AP site. Internally ^32^P-labeled DNA substrates containing a site-specific CPD (~ 200 bp in length) were incubated with six purified recombinant NER factors, and excised oligonucleotides containing both the ^32^P label and CPD were detected by denaturing polyacrylamide gel electrophoresis and the following autoradiography as described previously [12, 50]

The multi-step strategy for lesion recognition/verification described above provides novel insights into unprecedented potential of GG-NER in vivo. Once XPC could be stably attached to certain sites within the genome, GG-NER then acquires an opportunity to search around for relevant lesions, even though the lesions are not located exactly at the XPC-bound sites [3]. Possible XPC-anchoring sites may be generated by certain types of endogenous DNA lesions, for instance, apurinic/apyrimidinic (AP) sites, to which we have previously reported XPC in vitro can bind specifically [24]. In our cell-free NER system, a tetrahydrofuran, stabilized AP site analog inserted 5′ to a CPD indeed stimulates dual incisions around the CPD significantly (Fig. 2). XPC may be associated also with some DNA sites that are prone to be naturally unwound, especially under topological stresses in chromatin and/or during the processes of DNA replication and transcription. Although a substantial fraction of the damage search initiated in this way could be aborted eventually, such a mechanism may contribute as a kind of precautions against unpredictable occurrence of DNA lesions in the huge genome.

## UV-DDB and chromatin structures

It is no doubt that biochemical studies using cell-free systems have greatly contributed to our understanding of the GG-NER mechanism. However, most of those systems have utilized “naked” damaged DNA substrates of relatively small size. Considering situations in living cells, genomic DNA is so huge that the frequency of lesions generated under physiological conditions must be much lower in comparison with the in vitro systems. In addition, genomic DNA is organized into chromatin structures, which are quite heterogeneous and sometimes highly condensed, thereby preventing access to DNA binding protein factors. Therefore, one can reasonably assume existence of specialized molecular mechanisms, which assist XPC to discriminate its rare target sites efficiently from intact DNA present in large excess.

One of such mechanisms involves the UV-damaged DNA-binding protein complex (UV-DDB). This factor was identified as a heterodimer containing DDB1 and DDB2 proteins [25], which exhibits extraordinarily high binding affinity and specificity for UV-damaged DNA [26]. Its binding to pyrimidine (6–4) pyrimidone photoproducts (6–4PPs) is especially strong, whereas CPDs show moderate but significant affinity [27, 28]. In contrast, bulky base adducts induced by chemical mutagens are relatively poor substrates [29, 30]. Structural studies revealed that, unlike XPC, UV-DDB interacts directly with the UV-induced photolesions in the DNA duplex [31]. Accumulating evidence has established the notion that UV-DDB bound to a DNA lesion facilitates recruitment of XPC and the following initiation of NER [32–34]. This UV-DDB-mediated pathway is particularly important for efficient repair of CPDs, because XPC by itself poorly recognizes this type of lesion [35, 36].

In addition to recruitment of XPC, UV-DDB probably plays critical roles in the context of chromatin structures. Biochemical studies have shown that DNA lesions within the nucleosome core tend to refrain from interaction with XPC and the following repair reaction [37, 38]. In contrast, UV-DDB can interact with the nucleosome core containing DNA damage [39], suggesting that UV-DDB may induce alteration of chromatin structures such that DNA lesions are accessible to XPC. There have been a number of reports concerning interactions of UV-DDB with histone modification enzymes and/or chromatin remodeling factors. For instance, UV-DDB is part of a CUL4-RBX1 ubiquitin ligase (CRL4^DDB2^) [40, 41], which is activated upon binding to a DNA photolesion and ubiquitinates histones in addition to XPC and DDB2 [42–44]. Furthermore, tethering of UV-DDB to specific genomic loci induces relaxation of chromatin [45]. It is conceivable that global decondensation of chromatin would be advantageous for XPC to find lesions already marked by UV-DDB.

In striking contrast to CPDs, however, UV-induced 6–4PPs can be repaired quite efficiently even in the absence of UV-DDB [35, 36], most likely through direct recognition by XPC. Because 6–4PPs are shown to be generated by UV irradiation in chromatin in a relatively random manner [46], this fact strongly suggests existence of unprecedented molecular mechanisms that enable alteration of chromatin structures prior to lesion detection by XPC. We have recently reported that XPC physically interacts with histone H3 and this interaction is negatively regulated by acetylation of the histone protein [47]. Moreover, in living cells, inhibition of histone deacetylases compromises GG-NER, and deacetylation of histones appears to occur around sites with UV-induced DNA damage. Based on these findings, we propose that DNA damage associated with a relatively large helix distortion may be able to induce local reorganization of chromatin including deacetylation of histones, which may contribute to efficient recruitment of XPC [47]. Since chemical changes in DNA caused by lesions per se are only small, roles for chromatin structures could be completely different from the situation of UV-DDB-bound lesions.

**Fig. 3:**
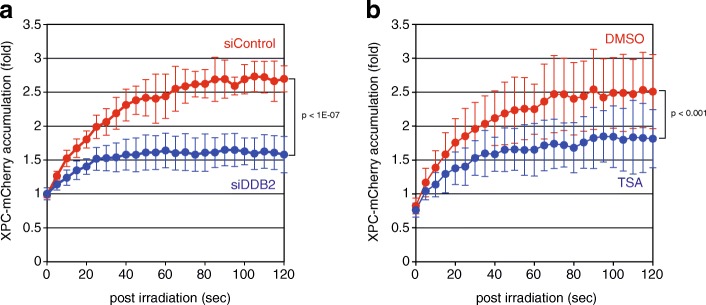
Kinetic analyses of XPC recruitment to DNA damage sites in living cells. DNA damage was induced with the 780-nm femtosecond laser and three-photon absorption within subnuclear regions of human osteosarcoma U2OS cells stably expressing mCherry-fused XPC. Cells were pre-treated either with siRNA targeting DDB2 (**a**) or with a histone deacetylase inhibitor, trichostatin A (TSA) (**b**). Time-lapse images were acquired every 5 s with the Olympus FV-3000 confocal laser scanning microscope, and relative fluorescence intensities of the irradiated areas were quantified. Mean values and standard deviations were calculated from 10 (**a**) and 17 (**b**) samples, respectively

In order to explore novel molecular mechanisms underlying regulation of DNA damage recognition for GG-NER, we have recently set up a confocal laser scanning microscopy equipped with a 780-nm femtosecond fiber laser, with which local DNA damage similar to that induced by 260-nm UV irradiation can be generated within cell nuclei by a principle of three-photon absorption. This system enables us to observe retarded accumulation of fluorescence-tagged XPC to the damaged sites after suppression of DDB2 expression or treatment with a histone deacetylase inhibitor (Fig. 3). Screening of siRNA and chemical compound libraries is ongoing with this system, which would shed light on new aspects of in vivo regulation of GG-NER.

## Conclusion

In eukaryotic GG-NER, simultaneous achievement of broad substrate specificity and accuracy relies on concerted actions of XPC, TFIIH, and XPA. Utilizing intrinsic sites that allow XPC binding, it is possible that this stepwise strategy further extends potential of lesion recognition in GG-NER. Although our biochemical results suggest a specific role of the 5′-3′ helicase of XPD especially in lesion verification, precise functions of the two ATPase/helicase subunits in TFIIH still remain to be established (e.g., which DNA strand each subunit interacts with and how the DNA duplex is locally unwound to form the pre-incision complex).

UV-DDB plays crucial roles in efficient recognition and repair of UV-induced DNA photolesions. Notably, effects of UV-DDB on the cell-free NER reaction have been somewhat controversial despite clear stimulation of GG-NER of UV-induced photolesions observed in vivo [43, 48, 49]. It is possible that such stimulation by UV-DDB may become more obvious when damaged DNA is packed into chromatin structures and/or when density of lesions within substrate DNA is lowered substantially. Furthermore, identification and addition of histone modifying enzymes and/or chromatin remodeling factors may be necessary to fully reconstitute both UV-DDB-dependent and independent forms of GG-NER. One of our goals would be to recapitulate the GG-NER processes with damaged chromatin substrates in the cell-free system.

## Abbreviations

6–4PP: Pyrimidine (6–4) pyrimidone photoproduct
CPD: Cyclobutane pyrimidine dimer
GG-NER: Global genomic nucleotide excision repair
NER: Nucleotide excision repair
TFIIH: Transcription factor IIH
UV: Ultraviolet light
UV-DDB: UV-damaged DNA-binding protein complex
XP: Xeroderma pigmentosum
